# Supplementary material for: Tunable Photodetectors via In Situ Thermal Conversion of TiS3 to TiO2
Source: Nanomaterials (Basel). 2020 Apr 9;10(4):711. doi: 10.3390/nano10040711 (PMC7221968; doi:10.3390/nano10040711)
Supplement: Supplementary file 1 [file nanomaterials-10-00711-s001.pdf]

Supporting Information: Tunable photodetectors via *in situ* thermal conversion of TiS_3 to TiO_2

Foad Ghasemi[1,2,+], Riccardo Frisenda*[3,+], Eduardo Flores[4], Nikos Papadopoulos[5], Robert Biele[6,7], David Perez de Lara[1], Herre van der Zant[5], Kenji Watanabe[8], Takashi Taniguchi[8], Roberto D'Agosta[6,9], Jose R. Ares[4], Carlos Sánchez[4,10], Isabel J. Ferrer[4,10] and Andres Castellanos-Gomez*[3]

[1] Instituto Madrileño de Estudios Avanzados en Nanociencia (IMDEA-Nanociencia), Campus de Cantoblanco, E-28049 Madrid, Spain.

[2] Nanoscale Physics Device Lab (NPDL), Department of Physics, University of Kurdistan, 66177-15175 Sanandaj, Iran.

[3] Materials Science Factory, Instituto de Ciencia de Materiales de Madrid (ICMM-CSIC), E-28049, Madrid, Spain.

[4] Materials of Interest in Renewable Energies Group (MIRE Group), Dpto. de Física de Materiales, Universidad Autónoma de Madrid, UAM, Campus de Cantoblanco, E-28049 Madrid, Spain.

[5] Kavli Institute of Nanoscience, Delft University of Technology, Lorentzweg 1, Delft 2628 CJ, The Netherlands.

[6] Nano-Bio Spectroscopy Group and European Theoretical Spectroscopy Facility (ETSF), Universidad del País Vasco CFM CSIC-UPV/EHU-MPC & DIPC, Av.Tolosa 72 ,20018, San Sebastián, Spain.

[7] Institute for Materials Science and Max Bergmann Center of Biomaterials, TU Dresden, 01062 Dresden, Germany.

[8] National Institute for Materials Science, Namiki 1-1, Tsukuba, Ibaraki 305-0044, Japan.

[9] IKERBASQUE, Basque Foundation for Science, 48013 Bilbao, Spain.

[10] Instituto Nicolás Cabrera, Universidad Autónoma de Madrid, UAM, Campus de Cantoblanco, E-28049 Madrid, Spain.

*E-mail: riccardo.frisenda@csic.es, andres.castellanos@csic.es.

[+] **These two authors contributed equally to this work.**

Section S1 – Additional optical characterization of the oxidation of TiS_3 nanoribbons

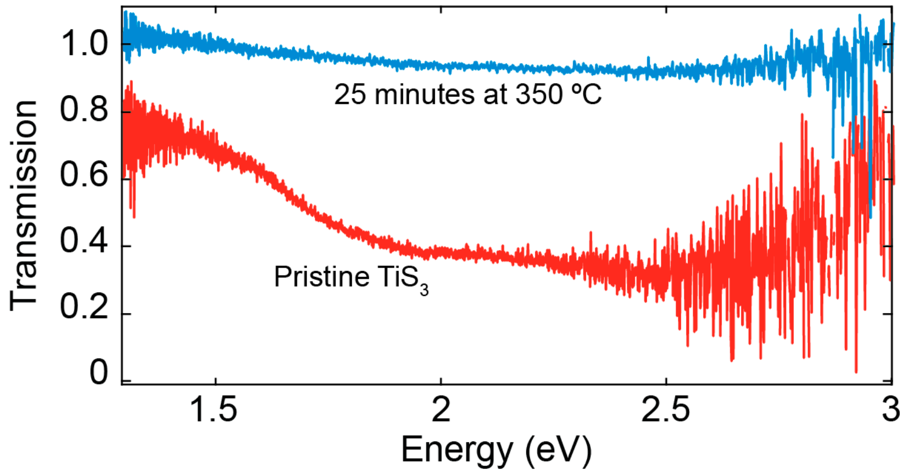

Figure S1: Energy resolved transmission of TiS_3 and TiO_2.

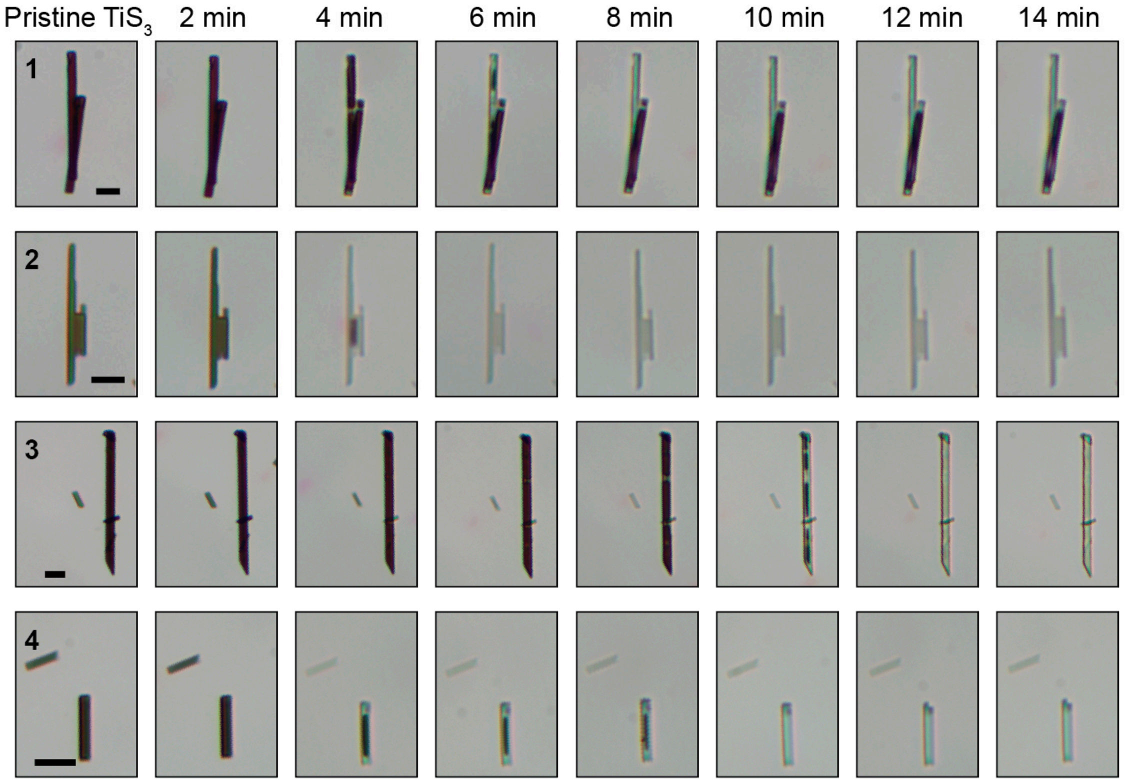

Figure S2: Optical image of individual TiS_3 nanoribbons transferred onto an ITO/glass substrate. The different rows show various nanoribbons identified in the same substrate. The leftmost picture of each row has been recorded on the pristine TiS_3, before heating the substrate. The subsequent images have been recorded after

heating the sample to 320 °C for the time indicated in each column. The black scale bars in the leftmost pictures correspond to 10 µm.

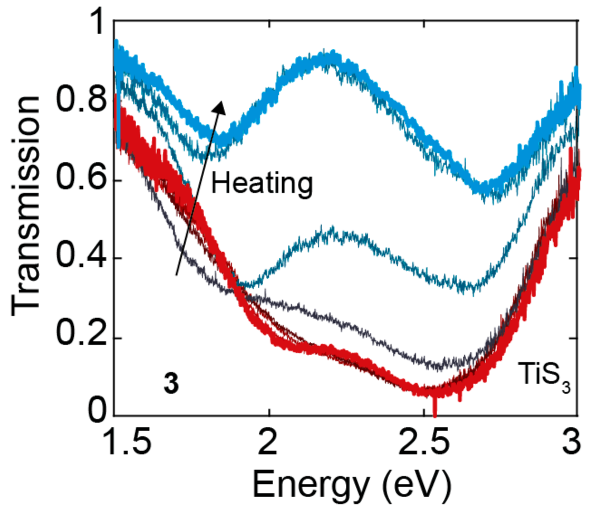

Figure S3: Energy resolved transmission of ribbons **3** and **4** from Fig. S2. The curves drawn with thick lines correspond to the initial (red) and final (blue) state of the ribbon under study.

Section S2 – TiS_3 photodetector annealed in vacuum

As a control experiment, to investigate the influence of the temperature in absence of oxygen, we studied a TiS_3 photodetector that we heated above 320 °C in vacuum. Figure S4a shows the responsivity of the device in its pristine state (red curve) and after 10, 20 and 30 minutes at 320 °C in vacuum. The shape of the responsivity (and the power exponent in panel b) is preserved and the device shows an almost flat response from less than 400 nm to more than 700 nm (as can be also seen in panel c), typical of TiS_3 photodetectors.

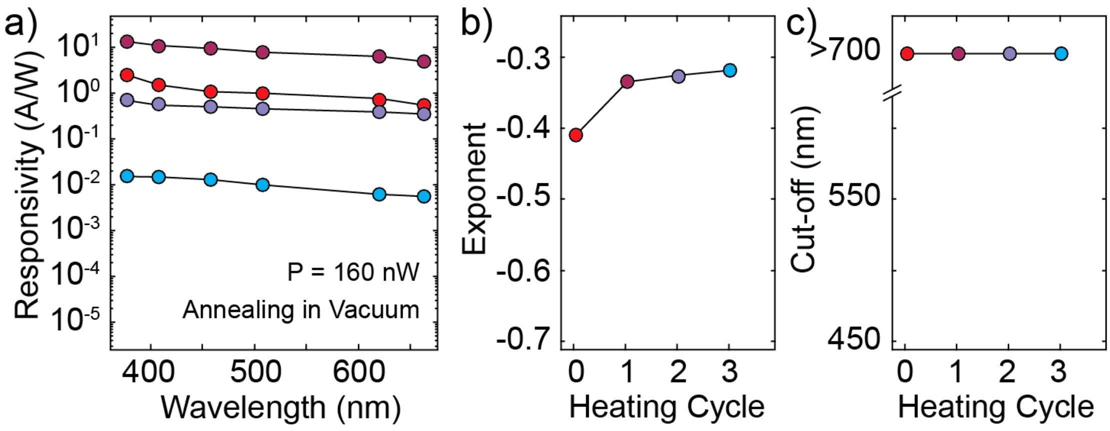

Figure S4: a) Responsivity of the device as a function of wavelength. The pristine TiS_3 device (top) was heated at 320 °C in vacuum in steps of 10 minutes and after each step the responsivity was measured. Note that the dark current decreases for

the successive heating cycles from 70 μA (pristine) to 20, 1.1 and 0.025 μA for the 1st, 2nd and 3rd heating cycle respectively. b) Responsivity exponent as a function of heating cycle. c) Cut-off wavelength as a function of heating cycle.

Section S3 –TiS_3 photodetectors annealed in air

In our study we characterized a total of 12 TiS_3 photodetecting devices, 11 devices have been heated above 320 °C in air (devices 1-11) and 1 device has been heated above 320 °C in vacuum as a control experiment. When heating up the device in air we managed to successfully convert the TiS_3 photodetector in a TiO_2 photodetector without losing the functionality in 4 devices, corresponding to a success rate of 36%. Figure S5a shows the responsivity as a function of wavelength of devices 1-11 each in its pristine state and Figure S5b shows the responsivity of devices 1-4, which have been successfully converted to TiO_2 photodetectors, in their initial and final states.

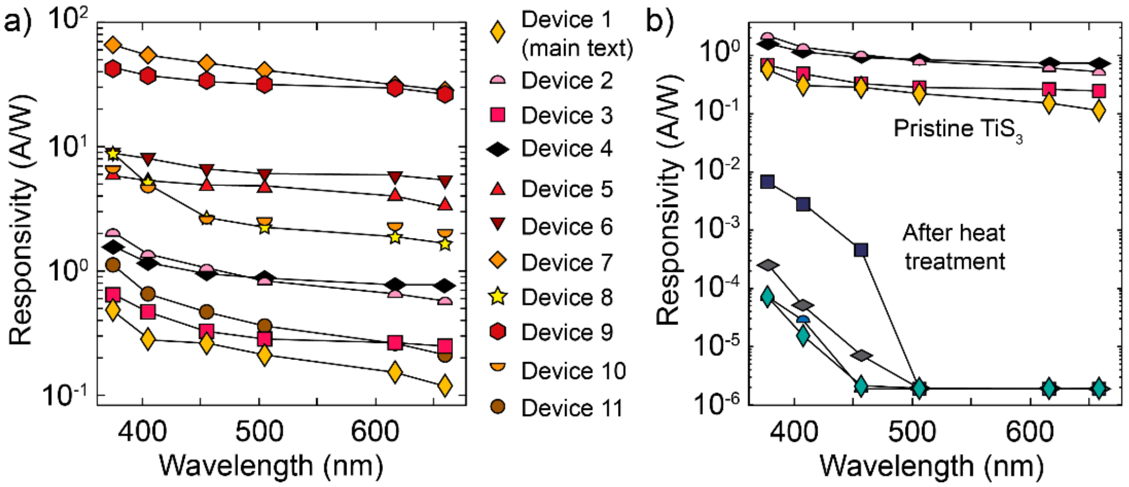

Figure S5: a) Responsivity as a function of wavelength of the pristine TiS_3 devices investigated in this work. b) Responsivity of the TiS_3 devices which have been successfully converted in TiO_2 photodetectors by heating at 320 °C. The four bottom curves correspond to the oxidized TiO_2 devices.

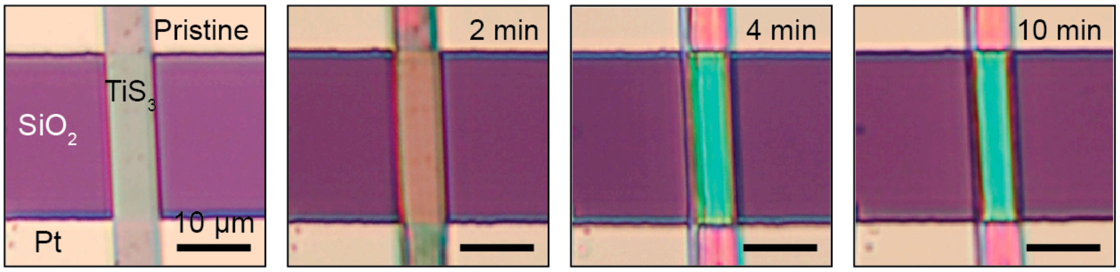

Figure S6: Optical microscope image of a TiS_3 photodetector (device 1) just after fabrication (left panel) and at different steps of the heating process.

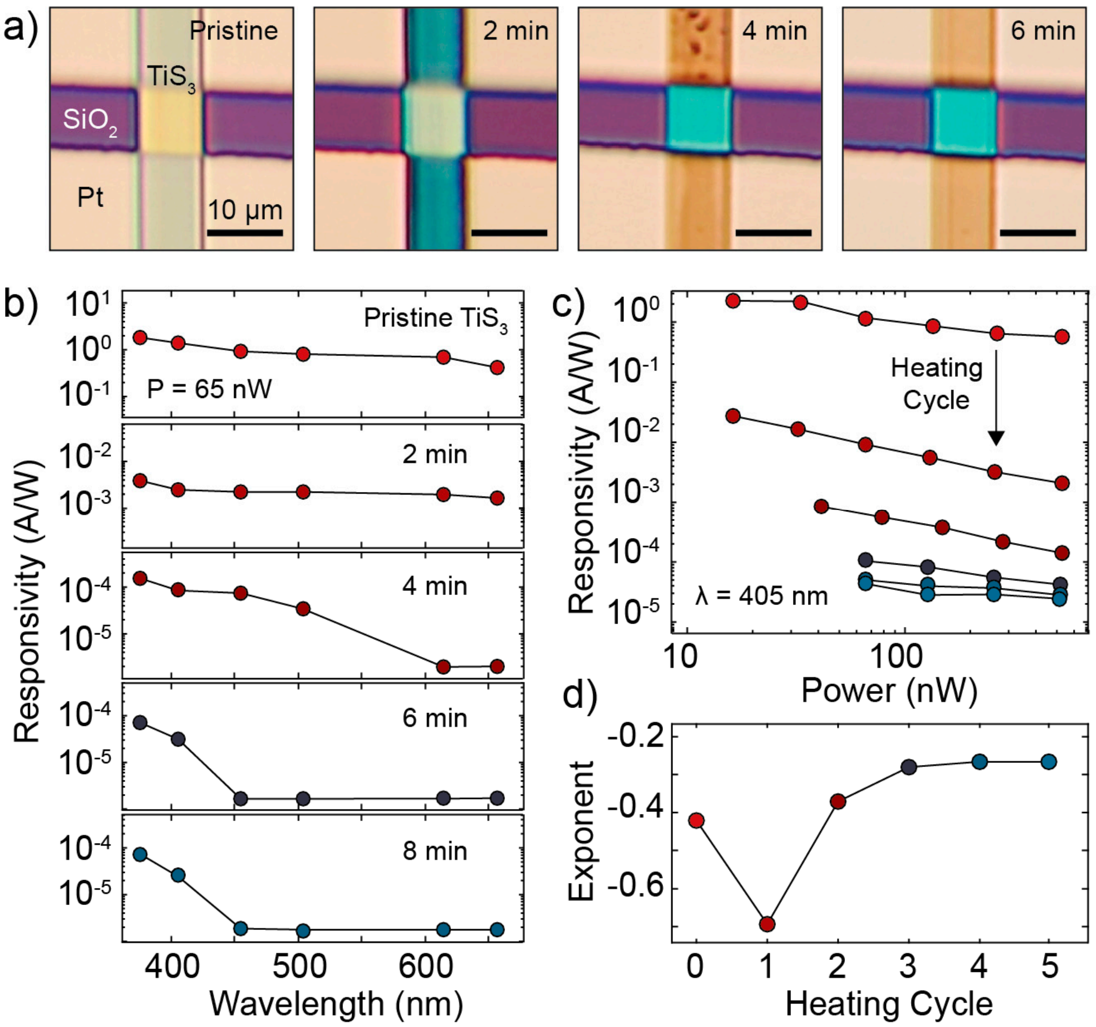

Figure S7: a) Optical microscope image of a TiS_3 photodetector (device 2) just after fabrication (left panel) and at different steps of the heating process. b) Responsivity of the device as a function of wavelength for different heating cycles. The pristine TiS_3 device (top) was heated at 320 °C in steps of 2 minutes and after each step the responsivity at different wavelengths was extracted. The two bottom curves correspond to the oxidized TiO_2 device. c) Responsivity of the device at 405 nm as a function of incident power for the pristine device (top curve) and after consecutive heating cycles. c) Responsivity-power law exponent as a function of heating cycle.

Section S4 –Stability of TiS_3 during Raman spectroscopy

We performed measurements of TiS_3 ribbons at various incident optical power densities and integration times to probe the stability of TiS_3 during the Raman spectroscopy experiments. The results, which are collected in Figs. S8 and S9, indicate that TiS_3 ribbons undergo photooxidation at an incident power of 10 mW (spot size ~2 um^2, power density 5 mW/µm^2). In the case of lower densities we do not observe any degradation or photooxidation for exposition times as large as 60 s.

Figure S8a shows a semilogarithmic representation of the Raman spectra of a TiS_3 ribbon recorded for different incident powers (in sequence 0.2 mW, 2 mW, 5 mW, 10 mW, 20 mW, 0.2 mW) and different integration times. In all the three panels one can see that the spectra recorded at the lowest excitation power of 0.2 mW in the pristine state and after the application of the larger powers are different. While the pristine spectrum (red) shows only the peaks due to TiS_3, the final spectrum shows an additional peak at 142 cm^{-1}, due to TiO_2, independent on the integration time. This indicates that the laser at 532 nm used in the Raman experiments can oxidize the TiS_3 ribbons. Figure S8b shows the spectra recorded with integration time 10 s with a vertical offset added for clarity. From these spectra it is clear that the additional peak at 142 cm^{-1} appears during the measurement at 10 mW. To quantify this phenomenon we perform a fit of the peaks at 142 cm^{-1} (due to TiO_2) and at 301 cm^{-1} (due to TiS_3) indicated by the shaded areas in panel b. Figure S8c shows the ratio between the areas of the peaks at 301 cm^{-1} and 142 cm^{-1} as a function of the incident power. By inspecting the plot we see that the ratio between the peaks is constant for powers as large as 5 mW (power density 2.5 mW/μm^2) and that rapidly decreases to zero (in an irreversible way) at a power of 10 mW (power density 5 mW/μm^2). In the case of an incident power equal or lower than 5 mW we do not observe any laser induced oxidation for integration times as long as 60 s as can be seen in Figure S9.

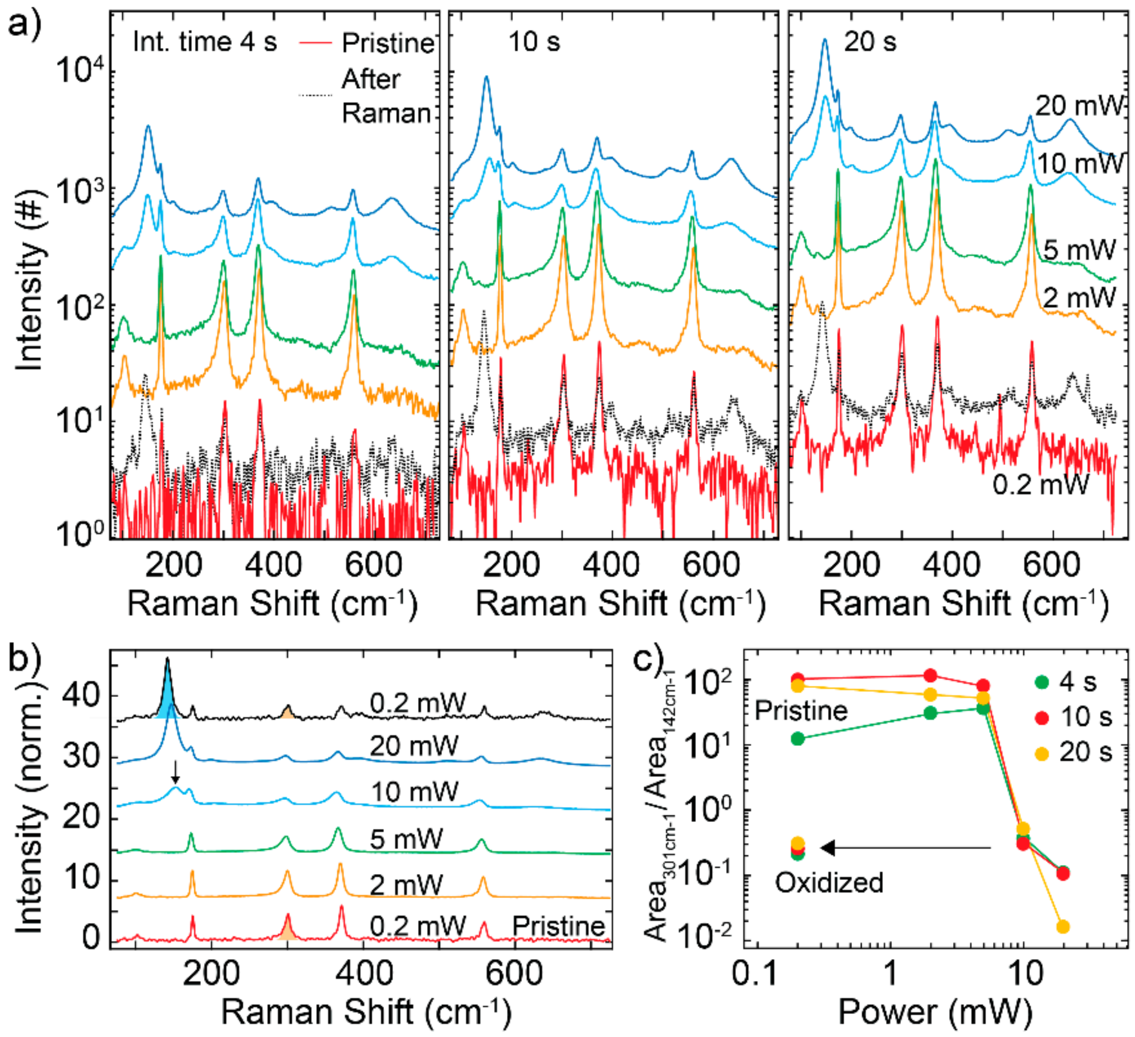

Figure S8: a) Raman spectroscopy of a TiS_3 ribbon as a function of illumination power (curves with different colours) and integration time. For each integration time (4 s, 10 s and 20 s) we select a different position in the ribbon to probe the pristine material. b) Raman spectra recorded with integration time 10 s at different incident powers. Each spectrum is normalized by the incident power. A vertical offset has been introduced for clarity. c) Ratio between the areas of the peaks at 301 cm^{-1} (related to TiS_3) and at 142 cm^{-1} (TiO_2) as a function of power.

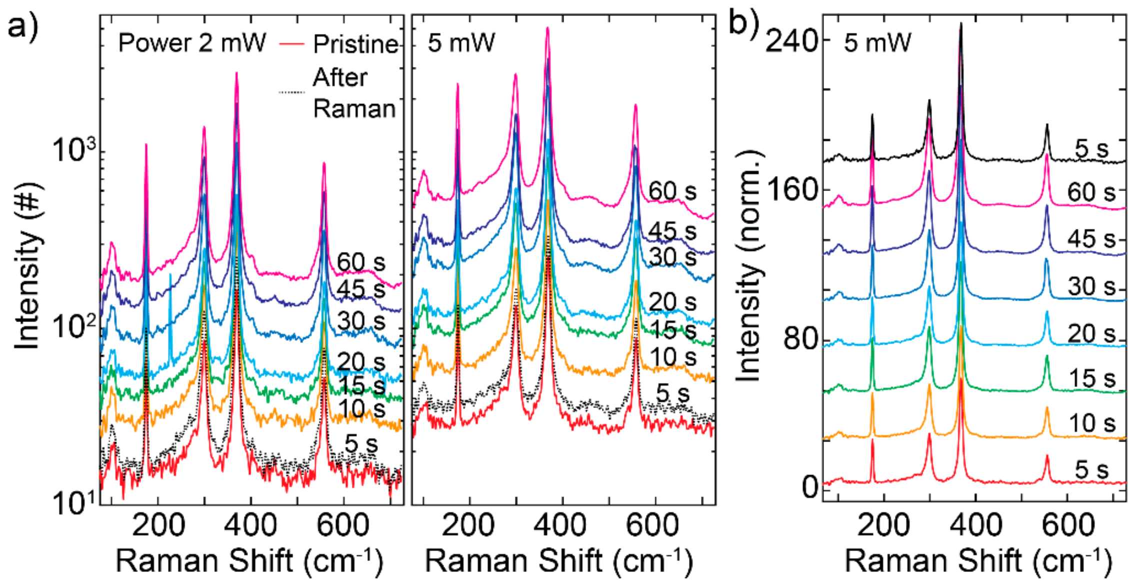

Figure S9: a) Raman spectroscopy of a TiS_3 ribbon as a function of integration time (curves with different colours) for different powers. For each power we select a different position in the ribbon to probe the pristine material. b) Raman spectra recorded with incident power 5 mW time at different integration times. Each spectrum is normalized by the integration time. A vertical offset has been introduced for clarity.
